# Karyotype characteristics, larval morphology and chromosomal polymorphism peculiarities of *Glyptotendipes salinus* Michailova, 1983 (Diptera, Chironomidae) from Tambukan Lake, Central Caucasus

**DOI:** 10.3897/CompCytogen.v10i4.9400

**Published:** 2016-11-04

**Authors:** Mukhamed Kh. Karmokov, Azamat Y. Akkizov

**Affiliations:** 1 Tembotov Institute of Ecology of Mountain territories RAS, I. Armand str., 37a, Nalchik 360051, Russia; 2 Institute of Biomedical Problems RAS, Center of Medico-Ecological Research, Shogentsukova str., 40, Nalchik 360051, Russia

**Keywords:** Diptera, Chironomidae, Glyptotendipes salinus, larval morphology, polytene chromosomes, chromosomal polymorphism, genetic distances, Tambukan Lake, central Caucasus (northern macroslope)

## Abstract

Data on the karyotype characteristics, larval morphology and features of chromosomal polymorphism of a population of *Glyptotendipes salinus* Michailova, 1983 (Diptera, Chironomidae) from Tambukan Lake (on the northern macroslope of the central Caucasus) are presented. It was found that diagnostic larval characters of *Glyptotendipes salinus* from Caucasus in general are similar to those described in previous studies, but with some significant differences. By some morphological characteristics Caucasian larvae appeared to be closer to *Glyptotendipes barbipes* than to ones provided for European larvae of *Glyptotendipes salinus* by Contreras-Lichtenberg (1999). Obtained morphological data make possible to conclude that Caucasian population of *Glyptotendipes salinus* can be a markedly diverged population of the species, probably even subspecies. In the Caucasian population 12 banding sequences were found: two in arms A, B, C, E, and G, and one in arms D and F. Eight of these are already known for this species, and four, salA2, salB2, salEX, and salG3, are described for the first time. Genetic distances between all the previously studied populations of *Glyptotendipes salinus* were measured using Nei criteria (1972). The population of the central Caucasus occupies a distinct position on the dendrogram compared with populations from Altai and Kazakhstan. All the obtained morphological and cytogenetic data can indicate the plausible relative isolation and complexity of the Caucasus from the viewpoint of microevolution. More researches are required in other parts of Caucasus and other geographically distant regions for more specific allegations.

## Introduction

*Glyptotendipes salinus* was first described by Michailova (1987) from Bulgaria. According to Fauna Europaea web source (http://www.faunaeur.org) the species is known in Europe from Austria, the British Isles and Bulgaria.

The karyotype of *Glyptotendipes salinus* has been studied from Bulgaria (Michailova 1987), Russia and Kazakhstan (Andreeva et al. 1998, Aimanova et al. 2000). In Russia this species is known from several regions (Altai Krai, Omsk Oblast, and Chelyabinsk Oblast). In Kazakhstan the two studied populations of the species are situated in the area of the Semipalatinsk Test Site (STS).

The species is a sibling species of *Glyptotendipes barbipes* that allows study of the earlier phases of divergence of the species in genus *Glyptotendipes* (Michailova 1987a, 1989). The species *Glyptotendipes salinus* differs from *Glyptotendipes barbipes* by chromosomal rearrangements in the chromosome arms A, C, D and E. Significant difference was also found in the amount and quality of the centromeric C- heterochromatin. Thus, some bands that were in the euchromatic state in *Glyptotendipes salinus* were in the heterochromatic state in *Glyptotendipes barbipes* (Michailova 1987b). The amount of centromeric DNA in the Ist, IInd and IIIrd chromosomes differs greatly between the two species (Michailova and Nikolov 1992). It was also found that C-heterochromatin of *Glyptotendipes barbipes* consists of two different types of C-bands: the dark ones at the periphery of the centromeres, which correspond to satellite II DNA, and pale C-bands corresponding to the satellite I DNA in the middle of the centromeric regions. Such heterochromatin differentiation was not expressed as prominently in the centromeric regions of *Glyptotendipes salinus* (Michailova 2014).

Michailova et al. (2002) also provide research on the effects of lead on the polytene chromosomes of *Glyptotendipes salinus*. They found that exposure to lead results in a decrease in the activity of the nucleoli (NOR) and Balbiani Rings (BRs).

Earlier it was shown that *Glyptotendipes salinus* occurs in brackish water, while sibling species *Glyptotendipes barbipes* prefers fresh water (Michailova 2014).

The aim of the work was to present the description of karyotype characteristics, larval morphology peculiarities and chromosomal polymorphism of *Glyptotendipes salinus* from Tambukan Lake (northern macroslope of the central Caucasus). Also it was very important to compare chromosomal polymorphism characteristics of *Glyptotendipes salinus* from the Caucasus with earlier studies.

## Methods

The fourth instar larvae of *Glyptotendipes* were used in the karyological study. The larvae were collected from one site of the central Caucasus: 17.05.13, 43°27.30'N; 43°09.75'E, southern shore of Tambukan Lake, altitude *ca.* 550 m a.s.l. Tambukan Lake is a lake with bitter salt water (salinity varies from 30 to 100 grams per liter) located in the northern macroslope of the central Caucasus, near the border of Stavropol Krai and the Republic of Kabardino-Balkaria of Russia. The lake’s surface area is 1.87 km^2^, and its depth ranges between 1.5–3.1 m. With regard to vertical zonation, the collection site belongs to the steppe zone (typification of the zone variants was given according to Sokolov and Tembotov 1989).

The morphological terminology follows Sæther (1980). Head capsule and body of 20 larvae were slide mounted in Fora-Berlese solution. The specimens have been deposited in Tembotov Institute of Ecology of Mountain territories RAS, Nalchik, Russia. The karyotype and chromosomal polymorphism has been studied in 63 larvae from the Tambukan Lake.

Larvae for karyotype analysis were fixed in ethanol-glacial acetic acid (3:1). Slides of the chromosomes were prepared using the ethanol-orcein technique (see Dyomin and Ilyinskaya 1988, Dyomin and Shobanov 1990). The banding sequences were designated according to the accepted rule specifying the abbreviated name of the species, symbol of chromosome arm, and sequence number, for example salC1, salC2, etc. (Keyl 1962, Wülker 1973). The identification of chromosome banding sequences was performed with the use of the photomaps of Michailova (1983) and Andreeva et al. (1998); chromosome mapping was performed according to Martin and Porter (1973) and Kiknadze et al. (1998), with corrections of Andreeva et al. (1998).

Microscope Carl Zeiss Axio Imager.A2 was used to study the chromosome slides. The software package STATISTICA 10 was used for statistical analysis (cluster analysis).

The following parameters were used for comparison of characteristics of chromosomal polymorphism: the number of banding sequences in a population, the percentage of heterozygous larvae, and number of heterozygous inversions per specimen. Genetic distances between populations were calculated according to Nei criteria (Nei 1972) on basis of the original data and data of Andreeva et al. (1998) on inversion polymorphism of the species in Russia and Kazakhstan.

## Results

**Figure 1.**
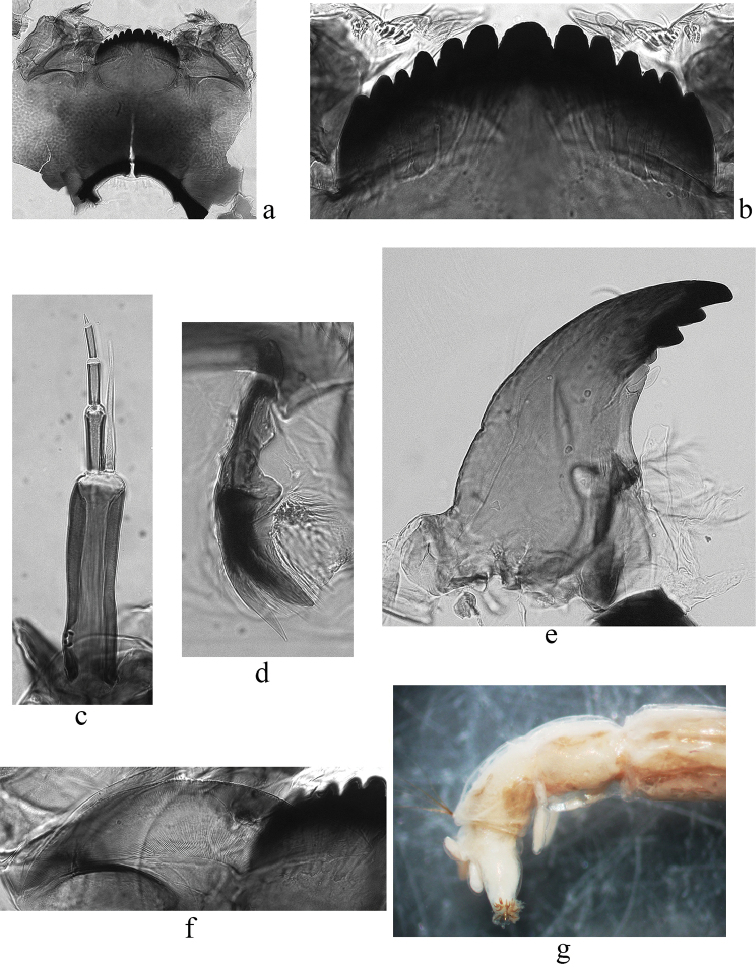
The larva of *Glyptotendipes salinus* from Tambukan Lake. **a** gular sclerite **b** mentum **c** antenna **d** premandible **e** mandible **f** ventromental plate **g** ventral tubuli on segment VIII.

The larvae of *Glyptotendipes* in the studied site were attributed to *Glyptotendipes salinus* by both morphological and chromosomal characteristics. Morphological characteristics of larva are presented in Fig. 1a–g.

The diagnostic larval characters of *Glyptotendipes salinus* from the Caucasian site in general are similar to those described previously for this species by Michailova (1987), Andreeva et al. (1998) and Contreras-Lichtenberg (1999), but there are some differences. The head capsule is lightly colored as in specimens found in other localities. Ventral tubuli are shorter than in *Glyptotendipes barbipes* and do not exceed the length of IX segment of larva body (Fig. 1g). The seta subdentalis of mandible is leaf-shaped (Fig. 1e), reaching the top of the last tooth. The ratio of the width of the ventramental plate to inter-plate distance (PSR) in *Glyptotendipes salinus* is more than 8 (8.34 according to Contreras-Lichtenberg (1999), from 8.02 to 9.04 in Caucasian population), while PSR of *Glyptotendipes barbipes* is 4.2. The width of the mentum of *Glyptotendipes salinus* was 219 µm according to Contreras-Lichtenberg (1999), but varies from 235 to 266 µm in Caucasian population, which is closer to *Glyptotendipes barbipes* measurements (256 µm). The ratio of the width of the mentum to the width of the middle tooth of mentum (MR) of *Glyptotendipes salinus* described by Contreras-Lichtenberg (1999) is 5.33, while in Caucasian population it varies from 6.8 to 7.17 and slightly exceeds MR=6.5 of *Glyptotendipes barbipes*. The width of the ventramental plate of *Glyptotendipes salinus* in Caucasian population varies from 297 to 340 µm (307.5 µm according to Contreras-Lichtenberg 1999), and its value is much higher than 288 µm of *Glyptotendipes barbipes*. The inter-plate distance (IPD) of *Glyptotendipes salinus* from Caucasian population is similar to data of Contreras-Lichtenberg (1999) – 36.9 µm – and varies from 34.5 to 37.5 µm, IPD of *Glyptotendipes barbipes* is much higher – 68 µm. The most significant difference was observed for the length of the larva’s body: in first description of Michailova (1987) and in paper of Contreras-Lichtenberg (1999) it is said to be 25-27 mm, while Caucasian larvae are twice shorter – 12-14 mm – which is very similar to an average larva’s length of *Glyptotendipes barbipes* (12 mm).

### Karyotype of *Glyptotendipes salinus* from the Central Caucasus

**Figure 2.**
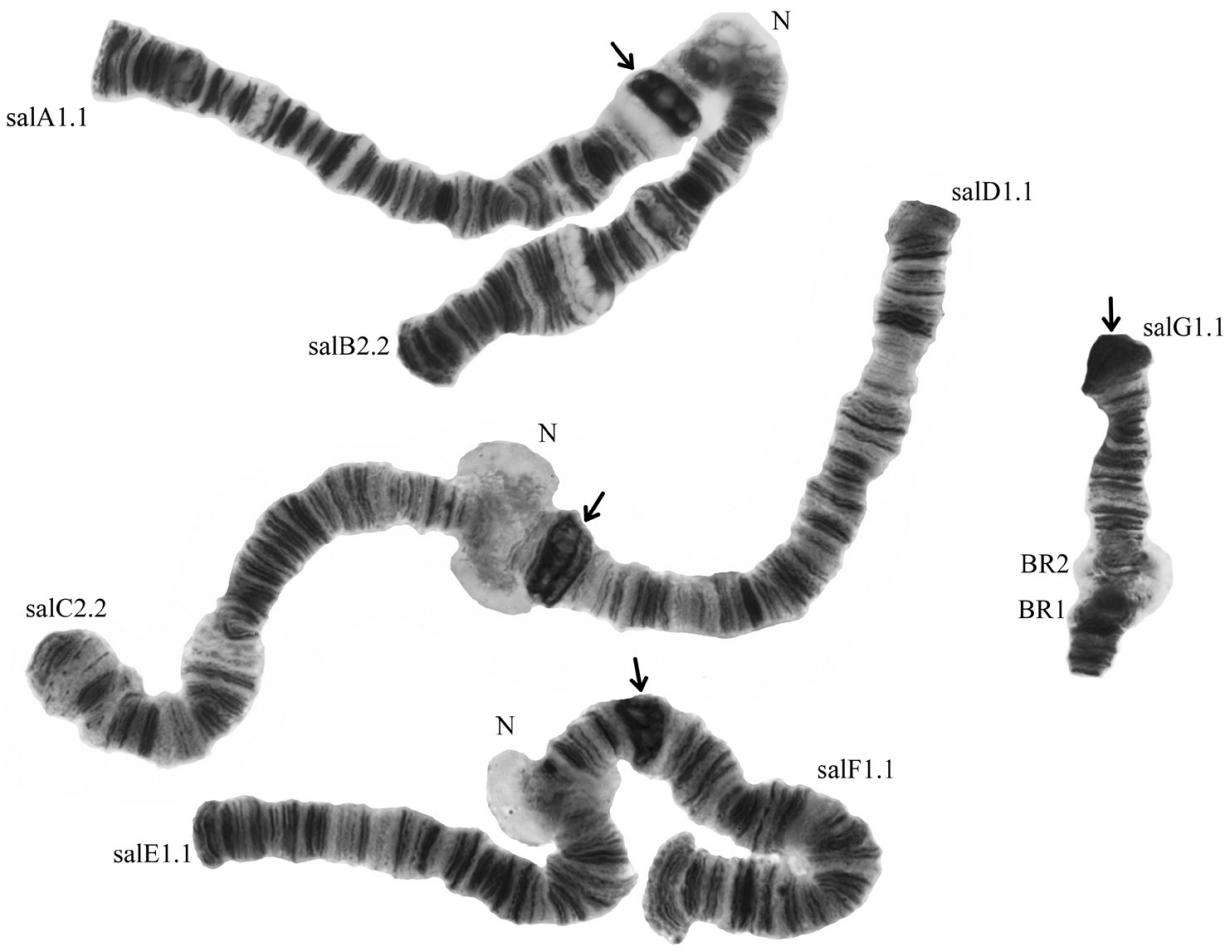
Karyotype of *Glyptotendipes salinus* from the Tambukan Lake; salA1.1, salD1.1 etc. – zygotic combinations of banding sequences; BR – Balbiani rings, N – nucleoli. Arrows indicate centromeric regions.

The diploid number of chromosomes in *Glyptotendipes salinus* karyotype is 2n = 8, chromosome arm combination is AB, CD, EF, and G (Fig. 2). Chromosomes AB and CD are metacentric, EF is submetacentric, and G is telocentric. Three well-developed nucleoli (N) are located on arms B, C, and E. There are two Balbiani rings (BR) in the karyotype: both are situated in arm G (Fig. 2).

The centromeric bands of long polytene chromosomes of *Glyptotendipes salinus* from the studied populations are large and belong to v-type according to the classification by Shobanov (2002).

### Banding sequences and chromosomal polymorphism of *Glyptotendipes salinus* from the Tambukan Lake

Until now, eleven banding sequences have been described in the banding sequences pool of *Glyptotendipes salinus* (Table 1). In the studied population only eight of those banding sequences were present, and four banding sequences have been found for the first time, providing a total of 12 banding sequences in the Caucasian population (Table 2).

**Table 1.** Catalog of banding sequences in the banding sequences pool of *Glyptotendipes salinus*.

Arm	Sequence	Order of bands	Authors of mapping
A	salA1	1a-b 5n-t 6a-n 2d-h 3ba 2u-i 3c-t 4a-v 5a-m 2cba 1t-n 1c-m 6o-t 7a-s	Andreeva et al.1983
	salA2	1ab 5n-t 6a-d 5h-a 4v-a 3t-c 2i-u 3ab 2h-d 6h-e 5i-m 2c-a 1t-n 1c-m 6o-t 7a-s	Original data
B	salB1	13z-a 12t-a 11o-a 10v-a 9n-a 8p-a 7c-a	Andreeva et al.1983
	salB2	13-z-j 9d-h 10a-v 11a-o 12a-t 13a-i 9cba 8p-a 7s-a	Original data
C	salC1	1a-o 4v-k 1p-r 2a-n 3a-p 4a-m 5a-z 6a-n 7a-i	Andreeva et al.1983
	salC2	1a-o 5f-a 4m-a 3p-a 2n-a 1r-p 4n-v 5l-z 6a-n 7a-i	Andreeva et al.1983
	salC3	1a-o 5f-a 4m-a 3p-a 2n-a 4u-n 1p-r 4v 5l-z 6a-n 7a-i	Andreeva et al.1983
	salC4	1a-d 4e-m 5a-k 1o-e 4d-a 3p-a 2n-a 1r-p 4n-v 5l-z 6a-n 7a-i	Andreeva et al.1983
D	salD1	12p-a 11m-a 10w-a 8a-g 8ih 8n-j 8o-q 9a-x 7i-a	Andreeva et al.1983
E	salE1	1a-u 3a-q 4a-w 5a-t 6a-r 2l-a 1v 6s-w 7a-l	Andreeva et al.1983
	salEX		Not mapped
F	salF1	11w-a 10s-a 9t-a 8m-a 7l-a	Andreeva et al.1983
G	salG1	5t-a 4i-a 3q-a 2r-a 1g-a	Andreeva et al.1983
	salG2	5t-a 4i 2b-r 3a-g 4a-h 2a 1g-a	Andreeva et al.1983
	salG3	5t-j 2h-r 3a-g 4a-i 5a-i 2g-a 1g-a	Original data

**Table 2.** Frequency of banding sequences in different populations of *Glyptotendipes salinus*.

Banding sequence	Populations					
	Kazakhstan		Altai Krai			Central Caucasus, Tambukan Lake (original data) 63 larvae
	STS, Atomnoe Lake (Andreeva et al. 1998) 50 larvae	STS, Shagan Lake (Andreeva et al. 1998) 52 larvae	Altai, Bulatovo Lake (Andreeva et al. 1998) 50 larvae	Altai, Gorkoe Lake (Andreeva et al. 1998) 49 larvae	Altai, Bolshoe Utichie Lake (Andreeva et al. 1998) 50 larvae	
salA1	1	1	1	1	1	0.992
salA2	0	0	0	0	0	0.008
salB1	1	1	1	1	1	0.317
salB2	0	0	0	0	0	0.683
salC1	0.220	0.164	0.630	0.622	0.530	0.016
salC2	0.780	0.817	0.370	0.378	0.470	0.984
salC3	0	0.009	0	0	0	0
salC4	0	0.009	0	0	0	0
salD1	1	1	1	1	1	1
salE1	1	1	1	1	1	0.992
salEX	0	0	0	0	0	0.008
salF1	1	1	1	1	1	1
salG1	0.950	0.991	1	1	1	0.968
salG2	0.050	0.009	0	0	0	0
salG3	0	0	0	0	0	0.032
Number of banding sequences in population	9	11	8	8	9	12
Percentage of heterozygous larvae	40	29	48	57	62	51
Number of heterozygous inversions per specimen	0.34	0.30	0.60	0.61	0.60	0.60

**Figure 3.**
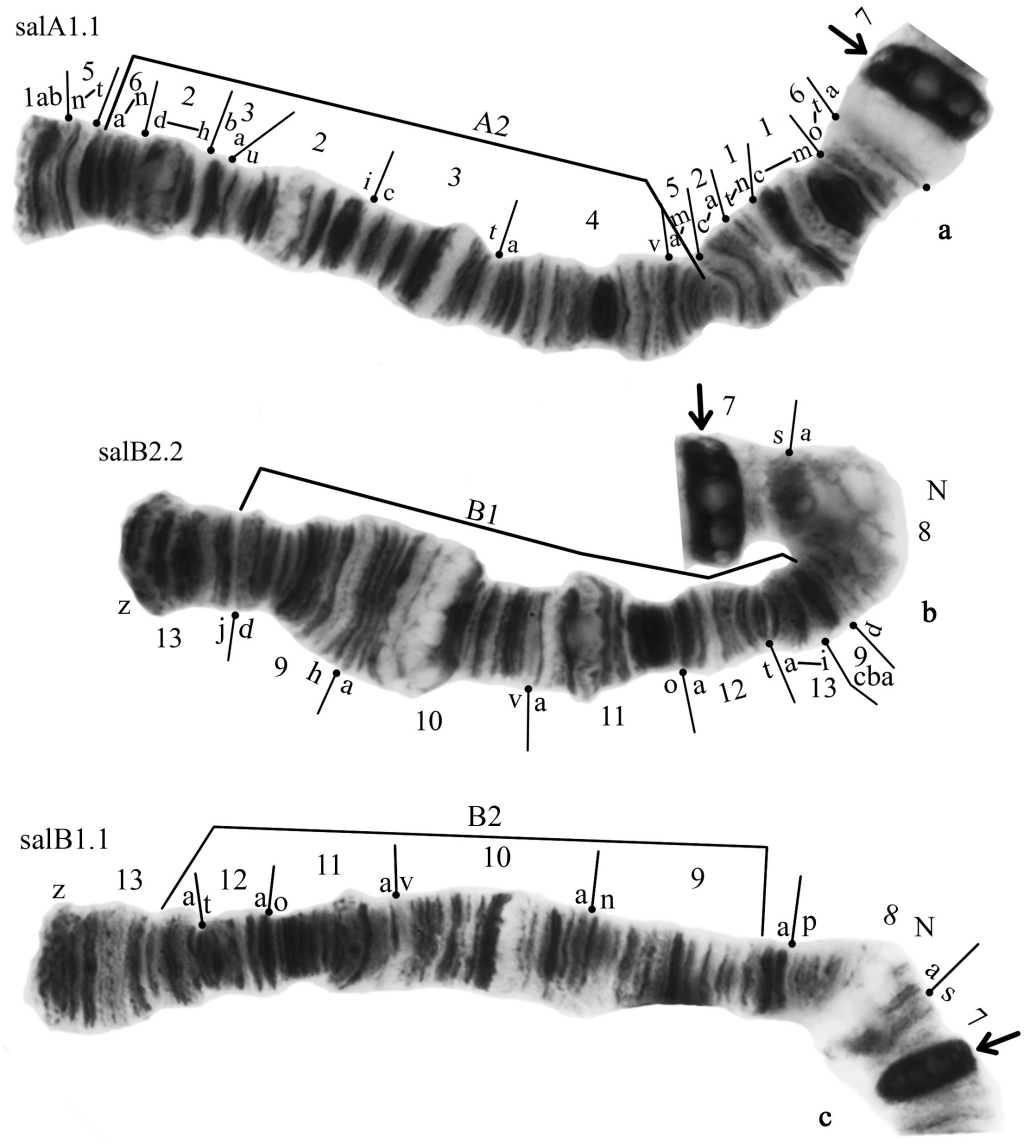
Banding sequences in arms A and B of *Glyptotendipes salinus*; **a** homozygotes salA1.1 **b** homozygotes salB2.2 **c** homozygotes salB1.1, Designations as in Fig. 2.

**Figure 4.**
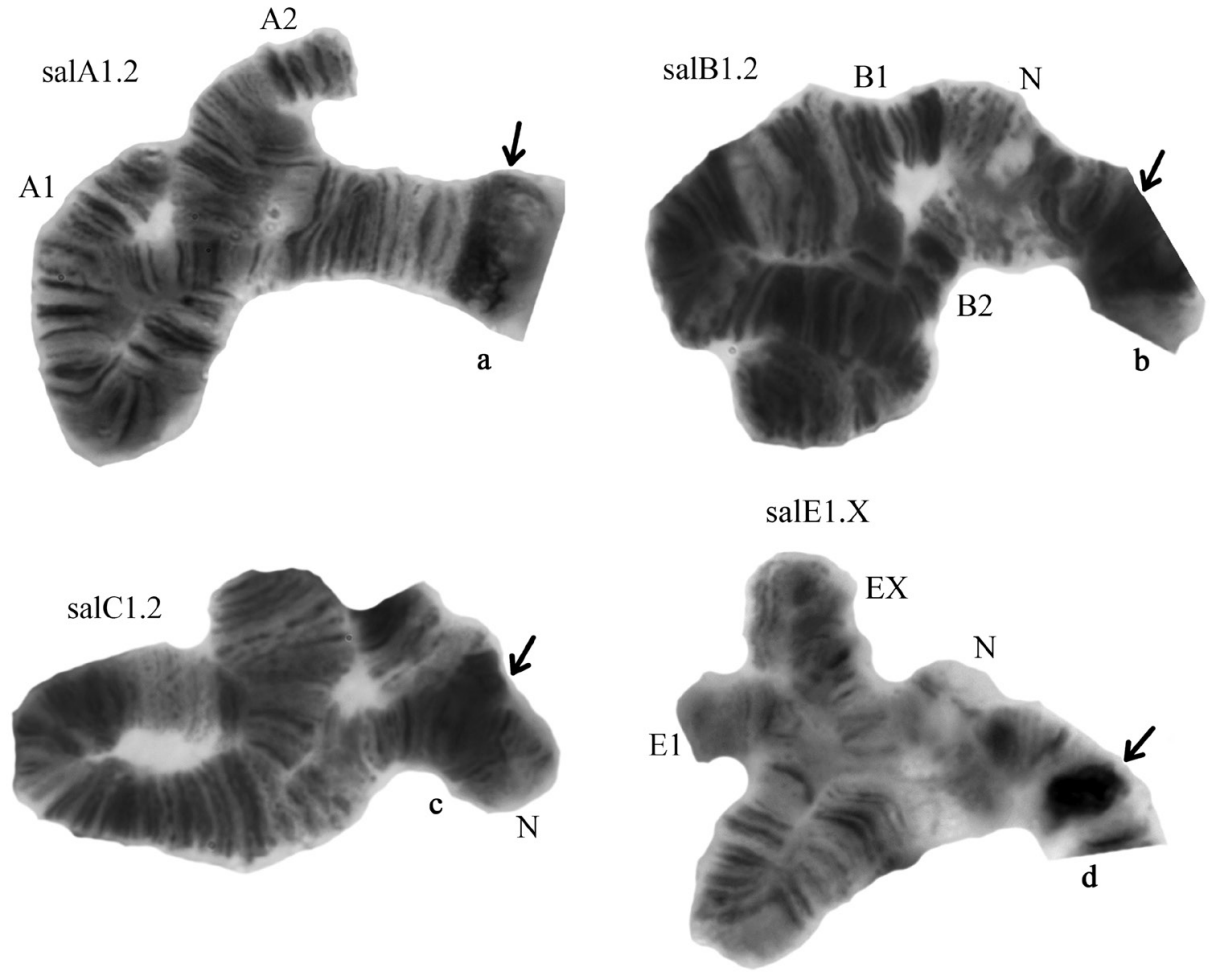
Chromosome inversions in different arms of *Glyptotendipes salinus* from Tambukan Lake. Heterozygous zygotic combination key: **a** salA1.2 **b** salB1.2 **c** salC1.2 **d** salE1.X. Designations as in Fig. 2.

**Arm A** has two banding sequences, salA1 and salA2 (Figs 3–4, Table 2). The banding sequence salA1 was predominant in the studied population (Table 2). The banding sequence salA2 was found only in the heterozygous state with very low frequency (salA1.1 – 0.968, salA1.2 – 0.032).

It is new for the species and described for the first time here (Fig. 4, Table 2). It differs from salA1 by one simple inversion that involves region 6e-n 2d-h 3ba 2u-i 3c-t 4a-v 5a-h:

salA2 1ab 5n-t 6a-d 5h-a 4v-a 3t-c 2i-u 3ab 2h-d 6h-e 5i-m 2c-a 1t-n 1c-m 6o-t 7a-s

**Arm B** has two banding sequences, salB1 and salB2 (Figs 3–4). The banding sequence salB2 was predominant in the studied population (Table 2). The sequence salB2 is new for the species and described for the first time (Figs 3–4, Table 2). It differs from salB1 by one simple inversion that involves region 13i-a 12t-a 11o-a 10 v-a 9h-d:

salB2 13-z-j 9d-h 10a-v 11a-o 12a-t 13a-i 9cba 8p-a 7s-a

The banding sequence salB2 was found with high frequency in both homozygous (salB1.1 – 0.095, salB2.2 – 0.445) and heterozygous states (salB1.2 – 0.460).

**Figure 5.**
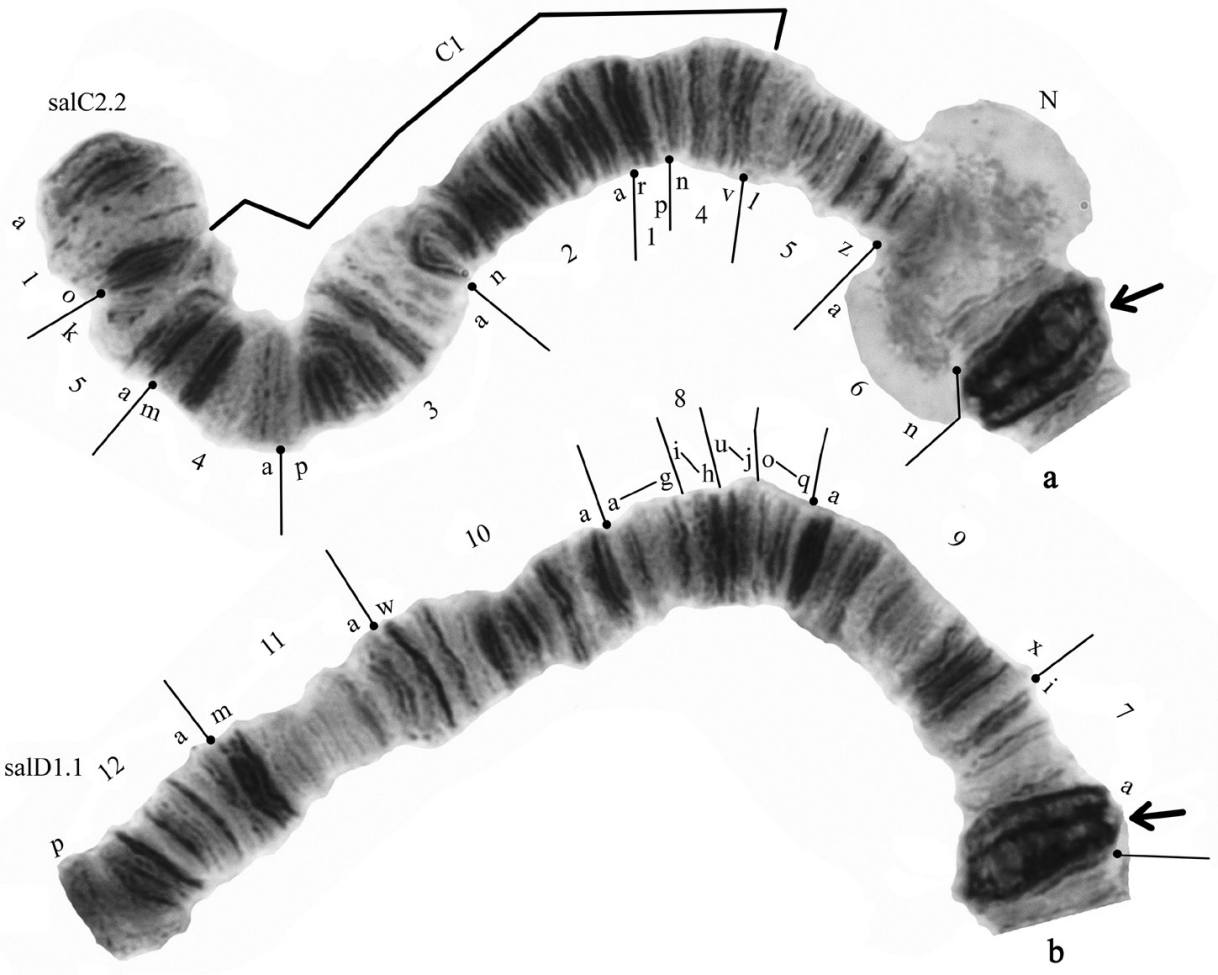
Banding sequences in the arms C and D of *Glyptotendipes salinus*. Key: **a** homozygotes salC2.2 **b** homozygotes salD1.1 Designations as in Fig. 2.

**Arm C** has two banding sequences, salC1 and salC2. The banding sequence salC2 was dominant in this population (Fig. 5, Table 2). The other banding sequence salC1 was found only in the heterozygous state (salC1.2 – 0.032, sal C2.2 – 0.968) (Fig. 4).

**Arm D** is monomorphic with banding sequence salD1 (Fig. 5, Table 2).

**Figure 6.**
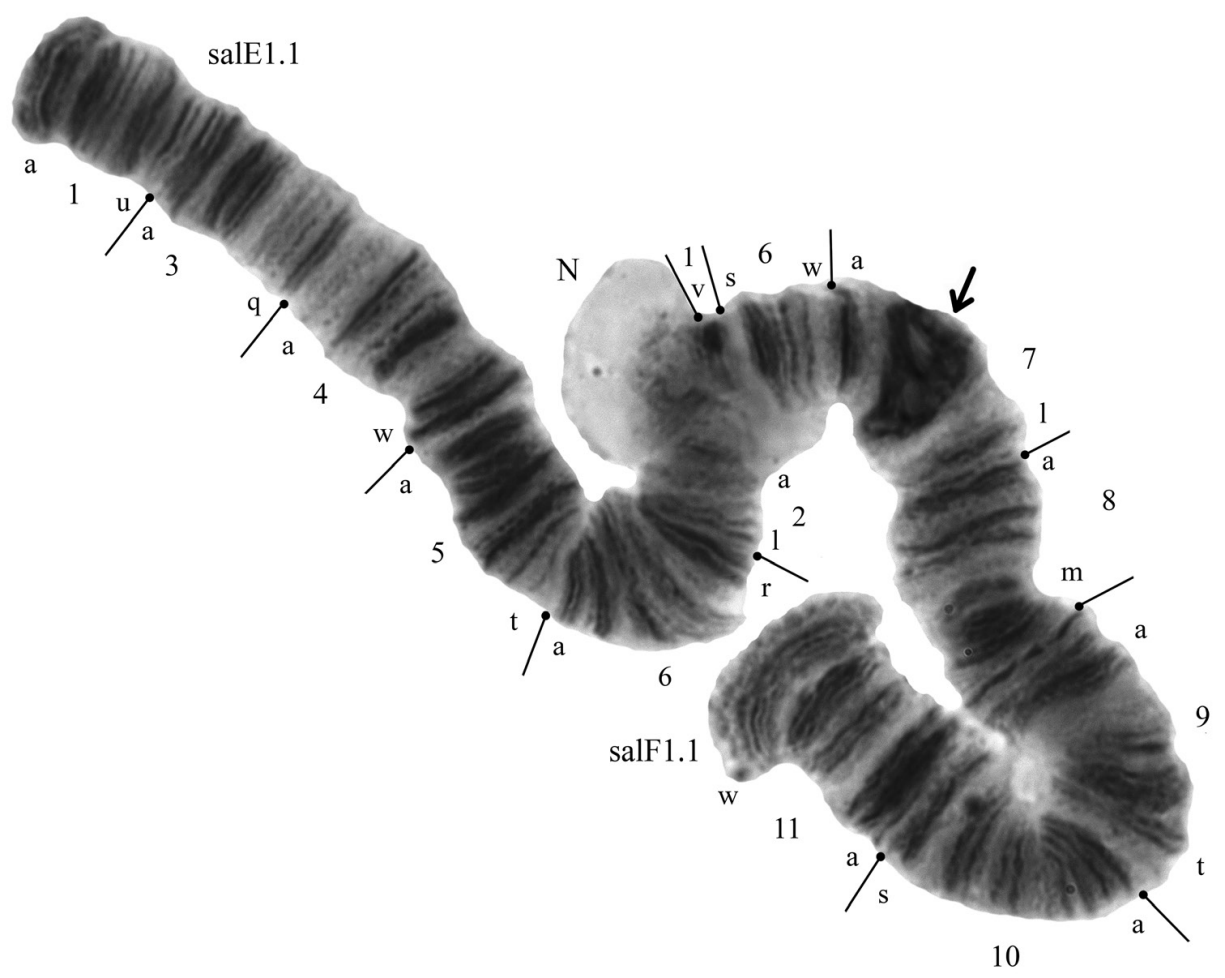
Banding sequences in the arms E and F of *Glyptotendipes salinus*. – The homozygotes salE1.1 and salF1.1. Designations as in Fig. 2.

**Arm E** had two banding sequences, salE1 and salEX (Table 2). The banding sequence salE1 was dominant in the population (Fig. 6, Table 2). The banding sequence salEX was found only in the heterozygous state (salE1.1 – 0.968, salE1.2 – 0.032). This banding sequence is new for the species and described here for the first time (Fig. 4, Table 2). Because banding structure of salEX was unclear it was impossible to map it and so no numerical designation was assigned to it.

**Arms F** is monomorphic with banding sequence salF1 (Fig. 6, Table 2).

**Figure 7.**
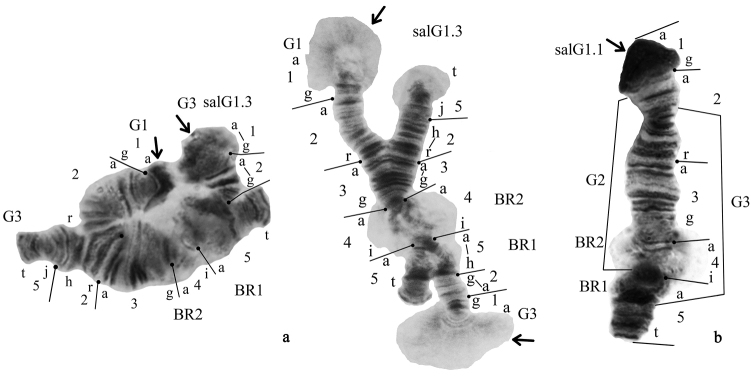
Banding sequences in the arm G of *Glyptotendipes salinus*. Key: **a** two different photo of heterozygote salG1.3 and **b** homozygote salG1.1. Designations as in Fig. 2.

**Arm G** had two banding sequences, salG1 and salG3. The banding sequence salG1 was dominant in the population (Fig. 7, Table 2). The banding sequence salG3 was found only in the heterozygous state (salG1.1 – 0.937, salG1.3 – 0.063). This banding sequence is new for the species and described for the first time (Fig. 4). It differs from salG1 by one simple inversion that involves region 5i-a 4i-a 3g-a 2r-h:

salG3 5t-j 2h-r 3a-g 4a-i 5a-i 2g-a 1g-a

### Comparison of chromosomal polymorphism of *Glyptotendipes salinus* from the Central Caucasus and other regions

Data for Russian (Altai Krai) and Kazakhstan populations are presented on the basis of publication of Andreeva et al. (1998).

**Arm A.** The populations from Altai and Kazakhstan (Andreeva et al. 1998) were characterized by the presence of single banding sequence in the arm, salA1 (Table 2). Same banding sequence is dominating in population from North Caucasus but one new for the species sequence salA2 was also found with very low frequency (0.008). The new banding sequence might be endemic for this region.

**Arm B** was monomorphic in populations of Altai and Kazakhstan and presented only by the banding sequence salB1 (Table 2). In the Caucasian population another banding sequence new for the species – salB2, was predominant. This new banding sequence is probably endemic for this region.

**Arm C** of *Glyptotendipes salinus* in all the studied populations was polymorphic. However in Altai populations the predominant banding sequence was salC1, whereas in Kazakhstan population dominated salC2. The population of the North Caucasus is closer to populations of Kazakhstan with salC2 dominating with even higher frequency (Table 2).

**Arm D** of *Glyptotendipes salinus* was monomorphic in all the studied populations.

**Arm E** was monomorphic in populations of Altai and Kazakhstan and low polymorphic in Caucasian population with the same dominant banding sequence salE1. A new banding sequence salEX was found in the Caucasian population with very low frequency (0.008) and might be endemic for the region.

**Arm F** of *Glyptotendipes salinus* was monomorphic in all the studied populations and presented only by the sequence salF1.

**Arm G** of *Glyptotendipes salinus* was monomorphic in populations of Altai and low polymorphic in populations of Kazakhstan and the Caucasus, although in all populations the dominant banding sequence was salG1. At the same time Kazakhstan and Caucasian populations differ by the set of rare inversions: salG2 was found in Kazakhstan while salG3 occurred in Caucasian population.

The inversion polymorphism of populations of *Glyptotendipes salinus* from the North Caucasus has a high level of heterozygous inversions per specimen and is similar to those of the Altai populations (Table 2). By the number of banding sequences per population (12), the Caucasian population is closer to Kazakhstan populations, but by the percentage of heterozygous larvae (51%) the studied population is more close to that of the Altai populations (48–62%).

**Figure 8.**
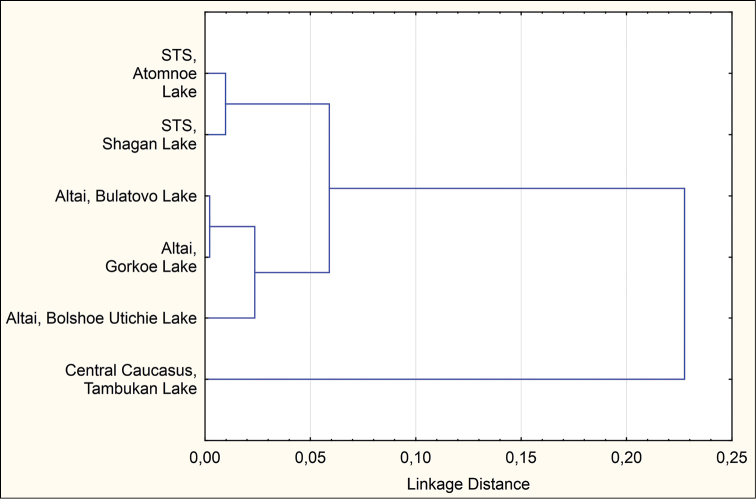
Tree dendrogram for six populations of *Glyptotendipes salinus*, single linkage, Euclidean distances.

Genetic distances (Table 3) measured by Nei criteria (1972) on the basis of original and previous data (Andreeva et al. 1998) on inversion polymorphism of the species in Altai region and Kazakhstan (Fig. 8) indicate the significant distance and distinct position of the Caucasian population of *Glyptotendipes salinus* in comparison with populations of Altai and Kazakhstan. The dendrogram was constructed on the basis of Nei criteria (1972) using NJ-method.

**Table 3.** Value of genetic distances between the different populations of *Glyptotendipes salinus*.

Population	STS, Atomnoe Lake	STS, Shagan Lake	Altai, Bulatovo Lake	Altai, Gorkoe Lake	Altai, Bolshoe Utichie Lake	Central Caucasus, Tambukan Lake
STS, Atomnoe Lake	0					
STS, Shagan Lake	0.00057	0				
Altai, Bulatovo Lake	0.02639	0.03204	0			
Altai, Gorkoe Lake	0.02539	0.03091	0.00001	0		
Altai, Bolshoe Utichie Lake	0.01519	0.01943	0.00153	0.00129	0	
Central Caucasus, Tambukan Lake	0.08167	0.07787	0.13945	0.13777	0.11999	0

## Discussion

In the northern Caucasus (central part of the northern macroslope) as well as in European Russia, *Glyptotendipes salinus* has been found for the first time.

As mentioned above, the diagnostic larval characters of *Glyptotendipes salinus* from the Caucasus in general are similar to those described by Michailova (1987), Andreeva et al. (1998) and Contreras-Lichtenberg (1999), but there are some significant differences as by some morphological characteristics Caucasian larvae of *Glyptotendipes salinus* are actually closer to *Glyptotendipes barbipes*. The data on larval morphology from the Caucasus are close to data provided by Contreras-Lichtenberg (1999) for *Glyptotendipes salinus* by PSR, width of ventramental plate and inter-plate distance (IPD). However, by width of mentum, MR and total length of larva body the Caucasian material is closer to *Glyptotendipes barbipes*. Also the length of the body of larva of *Glyptotendipes salinus* from Caucasian site is strikingly different from the data provided by Michailova (1987) and Contreras-Lichtenberg (1999) where it is said to be 25–27 mm, while Caucasian larvae are twice shorter (12–14 mm) and are very similar to *Glyptotendipes barbipes* (12 mm). Considering the data of Contreras-Lichtenberg (1999) the length of a larva body is the most different character of *Glyptotendipes salinus* in comparison to other species from the subgenus *Phytotendipes* Goethjebuer, 1934. The length of a larva body of *Glyptotendipes salinus* is the biggest (25–27 mm) among all other species: *Glyptotendipes pallens* (Meigen, 1804) – 10 mm, *Glyptotendipes glacus* (Meigen, 1818) – 12 mm, *Glyptotendipes gropekoveni* (Kieffer, 1913) – 13 (11–18) mm, *Glyptotendipes ospeli* Contreras-Lichtenberg, 1999 – 11 mm, *Glyptotendipes barbipes* (Staeger, 1839) – 12 m and *Glyptotendipes paripes* (Edwards, 1929) - 11–13 mm. At the same time the significant difference between larval length in previously described and Caucasian population of *Glyptotendipes salinus* suggest that further study of these species is necessary to determine the true characteristics of its larvae.

On the basis of morphological data one can conclude that the Caucasian population of *Glyptotendipes salinus* can be a markedly diverged population of the species, probably even subspecies. This conclusion is also supported by comparative analysis of inversion polymorphism between the Caucasian population and populations of other regions.

At present, 15 banding sequences including four new ones – salA2, salB2, salEX, and salG3 – are known in the banding sequences pool of *Glyptotendipes salinus*.

By frequencies of the banding sequences Caucasian population are closer to the Kazakhstan populations than to populations from Altai, but it clearly differ from populations from both other regions by the presence of four new banding sequences. The inversion polymorphism in population of *Glyptotendipes salinus* from the North Caucasus has a high level of heterozygous inversions per specimen and is similar to those of the Altai populations (Table 2). By the number of banding sequences per population, the Caucasian population is close to Kazakhstan populations, but in the percentage of heterozygous larvae, the studied population is more close to that of the Altai populations.

The population of the central Caucasus on the dendrogram of genetic distances (Fig. 8) occupies a distinct position while populations of Altai and Kazakhstan form their own clusters. All the obtained morphological and cytogenetic data may indicate the plausible relative isolation and complexity of the Caucasus from the viewpoint of microevolution. Such arrangement agrees rather well with the geographic location of the studied region. One can say that the north Caucasus is a relatively isolated territory, a special place, or a kind of “island” situated in the “sea” of steppes. Considering the presence in the Ciscaucasia and Greater Caucasus of a large number of saltwater lakes and rivers (Efremov et al. 2010), one can expect a large number of new records of this species in southern Russia. More researches are required in other parts of Caucasus, i.e. Western and Eastern Caucasus and other geographically distant regions for more specific allegations.
